# Predictive potential of tumour-stroma ratio on benefit from adjuvant bevacizumab in high-risk stage II and stage III colon cancer

**DOI:** 10.1038/s41416-018-0083-0

**Published:** 2018-05-14

**Authors:** Stéphanie M. Zunder, Gabi W. van Pelt, Hans J. Gelderblom, Christoph Mancao, Hein Putter, Rob A. Tollenaar, Wilma E. Mesker

**Affiliations:** 1 Department of Surgery, Leiden University Medical Centre, Albinusdreef 2, 2300 RC Leiden, Netherlands; 2 Department of Medical Oncology, Leiden University Medical Centre, Albinusdreef 2, 2300 RC Leiden, Netherlands; 3 Oncology Biomarker Development, Genentech Inc., CH-4070 Basel, Switzerland; 4 Department of Medical Statistics, Leiden University Medical Centre, Albinusdreef 2, 2300 RC Leiden, Netherlands

**Keywords:** Colon cancer, Tumour biomarkers

## Abstract

**Background:**

The tumour–stroma ratio (TSR) has proven to be an independent prognostic factor in colon cancer.

**methods:**

Haematoxylin eosin tissue slides of patients from the AVANT trial were microscopically scored for TSR and categorised as stroma -low or stroma -high. Scores were correlated to the primary and secondary endpoint disease-free survival (DFS) and overall survival (OS).

**Results:**

Patients with stroma-high tumours (*N* = 339, 28%) had a significantly shorter DFS (*p* < 0.001) compared to stroma-low tumours (*N* = 824, 68%). In the bevacizumab-FOLFOX-4 arm, DFS was significantly shorter compared to FOLFOX-4 in stroma-low tumours, with a hazard ratio (HR) of 1.94 (95% CI 1.24–3.04; *p* = 0.004). In stroma-high tumours a trend for better DFS was seen in bevacizumab-FOLFOX-4 vs. FOLFOX-4 (HR 0.61 (95% CI 0.35–1.07; *p* = 0.08)). For bevacizumab-XELOX vs. FOLFOX-4, this was not seen (stroma-low HR 1.07 (95% CI 0.64–1.77; *p* = 0.80); stroma-high HR 0.78 (95% CI 0.47–1.30; *p* = 0.35)). OS showed the same pattern for bevacizumab-FOLFOX-4 vs. FOLFOX-4 with a HR of 2.53 (95% CI 1.36–4.71; *p* = 0.003) for stroma-low and HR 0.50 (95% CI 0.22–1.14; *p* = 0.10) for stroma-high tumours. For bevacizumab-XELOX vs. FOLFOX-4, HR 1.13 (95% CI 0.55–2.31; *p* = 0.74) for stroma-low tumours and HR 0.74 (95% CI 0.37–1.51; *p* = 0.41) for stroma-high tumours.

**Conclusions:**

This exploratory analysis suggests a significantly shorter DFS and OS in stroma-low tumours with addition of bevacizumab to intravenous oxaliplatin-based chemotherapy, contrary to stroma-high tumours, where a beneficial trend is observed.

## Introduction

In Europe colorectal cancer (CRC) is the second most common cause of cancer related death in both men and women.^1^ The 5-year survival is strongly dependent on disease stage and rapidly decreases in individuals with lymph node or distant metastasis. Current guidelines for high-risk stage II and stage III patients, advice adjuvant fluoropyrimidine-based chemotherapy with addition of oxaliplatin as standard therapy. This combination has shown to significantly improve disease-free survival (DFS) and overall survival (OS).^2,3^ Adjuvant therapy with bevacizumab, a humanised anti-VEGF monoclonal antibody, has only demonstrated to improve outcome in patients with metastatic stage IV disease and is therefore currently not recommended in other stages.^3–8^ However, due to heterogeneity of colon cancer, one could argue that some subpopulations could possibly benefit from targeted therapy in an adjuvant setting. To identify such potential groups, predictive parameters are necessary. Currently most biomarkers focus on tumour cells. However, recently the “seed-and-soil” principle has been revisited, focusing on the tumour microenvironment as a major factor responsible for metastasis.^9,10^ Studies have shown that during cancer progression, the normal stromal host compartments transform, due to complex intercellular communication between surrounding stromal host cells and cancer cells, in which a cross-talk of signalling molecules between these compartments leads to an activated state with production of various cytokines and growth factors creating an area favouring cancer progression and invasion, thus illustrating the importance of intratumoural stroma.^11–14^ Consistent with this principle, it has been proven that in colon cancer, high amounts of intratumoural stroma are associated with poor survival compared to tumours with low amounts of stroma.^15–18^ This prognostic parameter is also known as the tumour–stroma ratio (TSR), and entails a simple microscopic quantification of the amount of intratumoural stroma on a tumour tissue slide, which is derived after surgical resection. It has been validated in multiple studies, thereby demonstrating the robustness and potential of this fairly simple, quick and cost-effective pathological technique.^15,17,18^ Since the prognostic quality of the TSR is clear, it is interesting to evaluate whether this parameter could also serve as a predictive marker to improve risk stratification of patients with high-risk stage II and III colon cancer, in order to determine if subpopulations could benefit from the VEGF antibody bevacizumab in an adjuvant setting. Our hypothesis was that patients with high stromal tumours would benefit from adjuvant bevacizumab, considering these tumours hold features promoting cancer progression and metastasis, hence possessing a more aggressive phenotype.^11,12,14^ To study this concept, we used the study population from the AVANT trial (BO17920), a prospective randomised trial studying the addition of bevacizumab to oxaliplatin-based chemotherapy in an adjuvant setting. This was a negative study, showing no prolongation of DFS and for OS even suggesting a potential detrimental effect when adding bevacizumab to the chemotherapy regime. We considered that if the TSR is able to identify patients that do benefit from bevacizumab in an adjuvant setting, it could serve as a selection tool to optimise adjuvant treatment outcomes in colon cancer. Therefore, the aim of this study was to investigate the predictive potential of TSR, by determining the effects on DFS and OS in patients with high-risk stage II and stage III colon cancer who received standard oxaliplatin-based chemotherapy with or without addition of bevacizumab.

## Patients and methods

### Study design

Available haematoxylin and eosin (H&E)-stained tumour slides from patients randomised in the AVANT trial were included in our analysis. Patients entering the AVANT trial had undergone potential curative treatment, including surgery (before randomisation) followed by adjuvant chemotherapy.

Inclusion criteria were histologically confirmed high-risk stage II or stage III colon carcinoma. The study had an open-label design, in which patients were randomly assigned 1:1:1 to one of the three treatment regimens: FOLFOX-4 for 24 weeks followed by observation for 24 weeks, bevacizumab-FOLFOX-4 or bevacizumab-XELOX for 24 weeks followed by bevacizumab monotherapy for 24 weeks. Patients were recruited in 330 centres in 34 countries. For detailed trial design, see de Gramont et al.^5^

For our study, archival material was used in an anonymised matter; therefore, no additional informed consent was needed.

### Histopathologic scoring

The TSR was determined in all patients from whom an H&E-stained formalin-fixed paraffin-embedded tissue slide from the primary tumour was available.

Pathological examination was performed as described by Mesker et al. (2007) (for detailed description see Appendix 1). Two investigators (S.Z., G.vP.) scored stromal percentage in a blinded manner. Scoring percentages were given per 10-fold (10%, 20%, etc.) per image field. For statistical analysis, we defined two groups: stroma -high (>50%) and stroma- low (≤50%) as determined a priori to have maximum discriminative power (Figure S1).^17,18^

### Statistical analysis

Statistical analysis was performed using SPSS software version 23.0. The primary endpoint, DFS, was defined as the time between randomisation and recurrence, new occurrence of colon cancer, or death from any cause. Alive and event-free patients at the clinical cut-off date were censored at the last date at which they were known to be disease-free and/or alive. The secondary endpoint, OS, was defined as time from randomisation to death. Patients who were still alive at the clinical cut-off date were censored at the date at which they were last confirmed to be alive.

Kaplan–Meier method and log rank test were used to analyse time-to-event endpoints. Intra-observer variability was tested using Cohen’s kappa coefficient.

Univariate and multivariable analyses were performed using Cox-regression analysis. For predictive analysis, a Cox proportional hazard model including an interaction term between treatment arms and TSR was used. The interaction test was used to test the null hypothesis that TSR is not predictive for response to bevacizumab. Parameters with a *p*-value less than 0.10 in the univariate analysis were included in multivariable analyses.

## Results

### Study population

In the AVANT trial, a total of 3451 patients were recruited between 2004 and 2007. We received a total of 1213 histological samples. After scoring all samples, baseline clinical patient information was used for analysis. Upon this, one patient was excluded due to the presence of stage IV disease at the time of randomisation. The final study population comprised 1212 patients, with respectively 405 (33.4%) patients in the FOLFOX-4 arm, 401 (33.1%) in the bevacizumab-FOLFOX-4 arm and 406 (33.5%) in the bevacizumab-XELOX arm. Patient characteristics were reasonably balanced between the different groups (Table 1). Considering our study population compromised only a selection of the total AVANT population, we compared our study population to the total AVANT population. There were no apparent differences in distribution between treatment arms, stage, gender and age. Noteworthy to mention, in the AVANT trial high-risk stage II patients were recruited solely for exploratory analysis. Efficacy (intention-to-treat (ITT)) analysis was only performed on stage III disease. Our study population consists of 205 (16.9%) high-risk stage II and 1007 (83.1%) stage III cases, which were both used in the analysis because both groups are considered as candidates for adjuvant chemotherapy according to current European guidelines.^19^

**Table 1:** Patient characteristics

	Total study population	Tumour–stroma ratio				
		Stroma low		Stroma high		
	*N* (%)	*N*	%	*N*	%	*p*-Value
Treatment						
FOLFOX-4	405 (33.4)	267	68	123	32	0.32
FOLFOX-4 + bevacizumab	401 (33.1)	284	73	103	27	
XELOX +bevacizumab	406 (33.5%)	273	71	113	29	
Gender						
Male	673 (55.5%)	453	70	195	30	0.43
Female	539 (44.5%)	371	72	144	28	
Age (years)						
≤50	278 (22.9%)	189	72	72	28	0.75
51–64	556 (45.9%)	379	71	152	29	
65–70	247 (20.4%)	166	69	75	31	
71–80	129 (10.6%)	88	69	40	31	
>80	2 (0.2%)	2	100	0	0	
Disease stage						
Stage II (high risk)	205 (16.9%)	136	69	61	31	0.54
Stage III	1007 (83.1%)	688	71	278	29	
Previous hypertension						
No	786 (64.9%)	545	72	208	28	0.12
Yes	426 (35.1%)	279	68	131	32	
KRAS mutation^a^						
Positive	445 (36.7%)	296	68	139	32	0.04
Negative	328 (27.1%)	226	70	95	30	
BRAF mutation^a^						
Mutation	78 (6.4%)	56	72	22	28	0.84
Wild type	994 (82.0%)	688	71	285	29	
MMR status^a^						
MSS	930 (76.7%)	631	69	281	31	0.01
MSI	121 (10.0%)	97	80	24	20	
CEA (ng/L)						
≤5.0	1171 (96.6%)	799	71	325	29	0.08
>5.0	28 (2.3%)	15	56	12	44	

*MMR status* mismatch repair status, *MSI* microsatellite instability, *MSS* microsatellite stable, *CEA* carcinoembryonic antigen.

^a^ Data not available from all patients

### Scoring tumour stroma ratio

Of 1212 evaluated patients, 339 (28.0%) were scored as stroma -high and 824 (68.0%) as stroma -low. Forty-nine (4.0%) samples could not be scored for TSR due to poor histological quality and were therefore excluded. These samples consisted either of too little tissue material to score (i.e. biopsies), exclusively muscle tissue and/or lymph node tissue. Cohen’s kappa coefficient revealed a good level of agreement in the classification. Cox regression interaction term for TSR and treatment arms showed a significant value for DFS (*p* = 0.005) and OS (*p* = 0.007) (Table S2).

### Disease-free survival

**Fig. 1:**
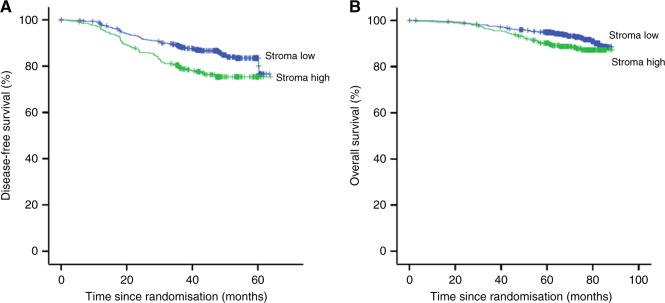
Kaplan–Meier survival curves of DFS (**a**) and OS (**b**) of stroma -low vs. stroma -high in the total patient population [DFS HR 1.75 (95% CI 1.32–2.33; *p* *<* 0.001)│OS HR 1.54 (95% CI 1.04–2.29; *p* = 0.03)]. Blue line indicates tumour stroma -low and green line tumour stroma -high. A full colour version of this figure is available at the British Journal of Cancer website

**Fig. 2:**
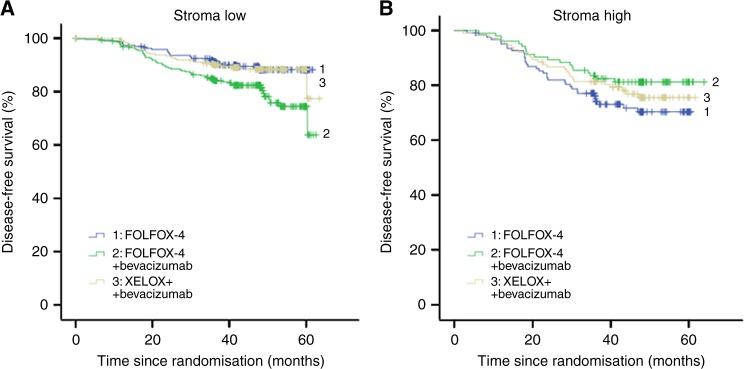
Disease-free survival: **a** stroma -low, **b** stroma- high. blue line—1: FOLFOX-4; green line—2: FOLFOX-4 + bevacizumab; grey line—3: XELOX + bevacizumab. A full colour version of this figure is available at the British Journal of Cancer website

DFS was significantly shorter in patients with stroma-high tumours compared to patients with stroma-low tumours, HR 1.75 (95% CI 1.32–2.33; *p* *<* 0.001) (Fig. 1). In the total BEP study population the addition of bevacizumab did not prolong the DFS (*p* = 0.23) compared to FOLFOX-4 monotherapy and suggests a potential detrimental effect on DFS (Figure S2). In the Cox-regression analysis, TSR had a HR of 2.92 (95% CI 1.78–4.79; *p* < 0.001) for the low vs. high stromal tumours. The interaction model for treatment arms and TSR showed a significant predictive value (*p* = 0.005) for treatment effect in the two TSR groups for DFS (Table S2). In the stroma-low group this effect was significant, with a HR of 1.94 (95% CI 1.24–3.04; *p* = 0.004) for bevacizumab-FOLFOX-4 vs. FOLFOX-4. For bevacizumab-XELOX this was not seen, with a HR of 1.07 (95% CI 0.64–1.77; *p* = 0.80). In the stroma-high tumours a trend for better DFS outcome was seen in the bevacizumab-FOLFOX-4 group vs. FOLFOX-4 (HR 0.61 (95% CI 0.35–1.07; *p* = 0.08). For bevacizumab-XELOX vs. FOLFOX-4 this was not seen (HR 0.78 (95% CI 0.47–1.30; *p* = 0.35)) (Table S2, Fig. 2). The univariate Cox regression analysis revealed TSR (*p* < 0.001), gender (*p* = 0.05*)*, disease stage (*p* = 0.002) and MMR status (*p* = 0.04) as statistically significant prognosticators for DFS. In the multivariable analysis TSR (*p* = 0.003), gender (*p* = 0.013) and disease stage (*p* = 0.004) maintained significance (Table S1).

**Fig. 3:**
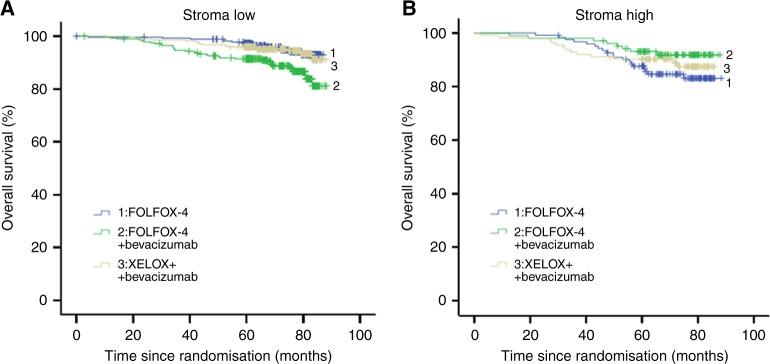
Overall survival: **a** stroma -low, **b** stroma -high. blue line—1: FOLFOX-4; green line—2: FOLFOX-4 + bevacizumab; grey line—3: XELOX + bevacizumab. A full colour version of this figure is available at the British Journal of Cancer website

#### Overall survival

As shown in Fig. 1, patients with stroma-high tumours had a significant shorter OS compared to patients with stroma-low tumours (HR 1.54 (95% CI 1.04–2.29; *p* =  0.03)). In the total BEP study population, the addition of bevacizumab did not prolong the OS (*p*  =  0.17) compared to FOLFOX-4 monotherapy (Figure S2). Cox-regression analysis for OS showed a HR of 3.14 (95% CI 1.57–6.26; *p*  =  0.001) for TSR with regard to high vs. low stromal tumours. The interaction model showed a similar pattern as for DFS, with a significant interaction term between treatment and TSR-group (*p*  =  0.007) (Table  S2). Stroma-low tumours in the bevacizumab-FOLFOX-4 arm vs. FOLFOX-4 arm had a significant worse OS, HR of 2.53 (95% CI 1.36–4.71; *p*  =  0.003). For stroma-high tumours this was not significant, with a HR of 0.50 (95% CI 0.22–1.14; *p*  =  0.10). For bevacizumab-XELOX vs. FOLFOX-4 the HR was 1.13 (95% CI 0.55–2.31; *p*  =  0.74) for stroma-low tumours and HR 0.74 (95% CI 0.37–1.51; *p*  =  0.41) for stroma-high tumours (Table  S2, Fig. 3). The univariate analysis for OS showed TSR (*p*  =  0.03), gender (*p*  =  0.006), disease stage (*p*  =  0.04) and BRAF status (*p*  =  0.10) as statistically significant prognosticators. In the multivariable analysis TSR (*p*  =  0.05), gender (*p*  =  0.002) and disease stage (*p*  =  0.05) maintained significance (Table  S1). No additional exploratory analyses were performed on patients from whom molecular variables were available (i.e. MMR status, KRAS and BRAF), due to non-significance in the Cox-regression analysis.

## Discussion

In our study, we evaluated the predictive potential of TSR in hopes of being able to select subpopulations with high-risk stage II and III colon cancer that could benefit from adjuvant bevacizumab. Prior research failed to show benefit from addition of bevacizumab to standard chemotherapy regimens in these patients and is therefore currently only recommended in metastatic disease.^4–8,20^ Our hypothesis was that high-risk stage II and III patients with high stromal tumours would benefit from adjuvant bevacizumab, considering the pro-carcinogenic features these tumours possess and association with a worse survival.^15–18,21^ In our study the TSR validated as a predictive parameter, however without clinical implications. As assumed, the stroma-low group had no benefit whatsoever from addition of bevacizumab and even showed a significantly detrimental effect on survival, most pronounced in the bevacizumab-FOLFOX-4 group. This was in accordance with the AVANT ITT analysis and supports current guidelines which discommend adjuvant anti-VEGF in stage II/III disease. It is not completely understood why this was so evident in this group and not as pronounced in the XELOX group. Considering capecitabine is biotransformed into active metabolites that mimic 5-FU infusion, one could consider these biologically equivalent and of similarly efficacy when administrated correctly.^22^ Previous studies investigating non-inferiority of capecitabine in combination with oxaliplatin vs. 5-FU with oxaliplatin, correspondingly showed either similar efficacy or inconclusive results regarding non-inferiority.^23–27^ The NO16966 accordingly showed similar performance of XELOX and FOLFOX in terms of OS, when adding bevacizumab.^28^ Taking this into account, it would be less likely to regard the observed results as due to an interaction of FOLFOX with bevacizumab. The AVANT ITT analysis does show considerably less adverse events, doses reductions, delays or interruptions in the XELOX group compared to the other groups, suggesting less toxicity and perhaps therefore better survival outcomes (for details, see de Gramont et al.^5^). However, since the ITT analysis only entails stage III patients, these results have to be adjusted for stage before correlation to our cohort is possible. In contrast with low stromal tumours, in patients with stroma-high tumours we did observe a beneficial trend with addition of bevacizumab. Although not significant, this was an anticipated effect when regarding high stromal tumours as more aggressive due to the cross-talk between their local microenvironment and tumour cells. This finding, in combination with previous research validating the TSR as an independent prognostic parameter, does suggest that there could be potential in the TSR as a predictive tool with clinical implications,^15,17,18^ perhaps not solely with TSR, but in combination with additional markers.^29^ However, that would compromise the simplicity and costs effectiveness of the current technique, which could be easily incorporated in routine diagnostics. Currently extensive research is being performed regarding the tumour microenvironment and response to anti-angiogenic therapy. It has become increasingly clear that stromal cells not only provide a target for cancer therapy, but also have an essential role in anti-angiogenic resistance.^30^ An issue, which is already relevant to patient groups receiving these agents in routine clinical practice, since benefit on OS with addition of bevacizumab is often borderline significant or lacking depending on the chemotherapy regimen.^31–33^ Better understanding of these mechanisms will make it possible to identify sensitive targets and/or phenotypes to overcome these tumour escape mechanisms. For instance, Smith et al. reported two stromal phenotypes (i.e. tumour-vessel and stromal-vessel) based on CD31 and α-smooth muscle actin (α-SMA) staining. In mCRC, tumour-vessel phenotype tumours appeared to be more sensitive to combination oxaliplatin-based chemotherapy with bevacizumab compared to the stromal-vessel phenotype.^34^ It would be interesting to correlate these phenotypes to the TSR, to possibly improve the predictive performance, but also to determine whether there is any prognostic relevance in metastatic disease. A possible limitation of this study is the fact we only investigated a selection of the total AVANT study population, though evenly balanced, making it possible that the study is underpowered. Nevertheless, despite the fact the findings were non-significant, we do find the potential beneficial survival trend that was observed in the stroma-high tumours with addition of bevacizumab, is worthwhile for further investigation with or without additional markers. Since this is one of the first studies evaluating this principle, we feel that we should not abandon this principle right away and validation of the findings would be necessary, to definitely rule out a coincidental finding. Considering very limited new targeted therapies have come available for treatment of colorectal cancer after the introduction of bevacizumab over a decade ago, maximum efficient utilisation of this drug would be desirable.

## Electronic supplementary material

Figure S1

Figure S2

Table S1

Table S2

Appendix 1
