# Supplementary figures and images for: Predictive potential of tumour-stroma ratio on benefit from adjuvant bevacizumab in high-risk stage II and stage III colon cancer
Source: Br J Cancer. 2018 May 14;119(2):164–9. doi: 10.1038/s41416-018-0083-0 (PMC6048031; doi:10.1038/s41416-018-0083-0)

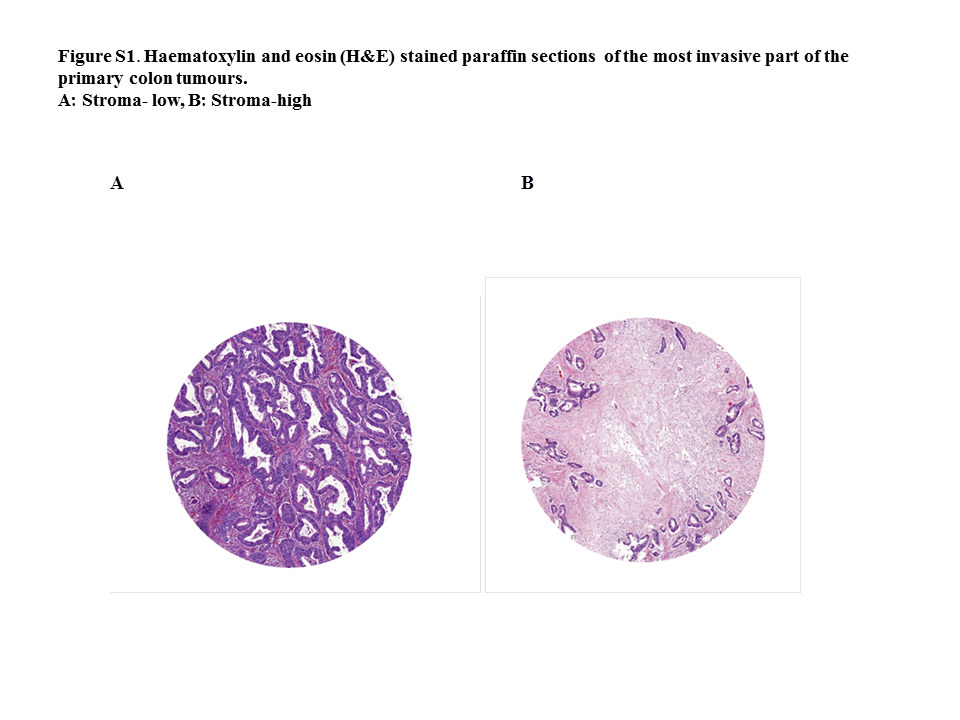

Supplement: Supplementary file 1 — Figure S1 [file 41416_2018_83_MOESM1_ESM.tif]

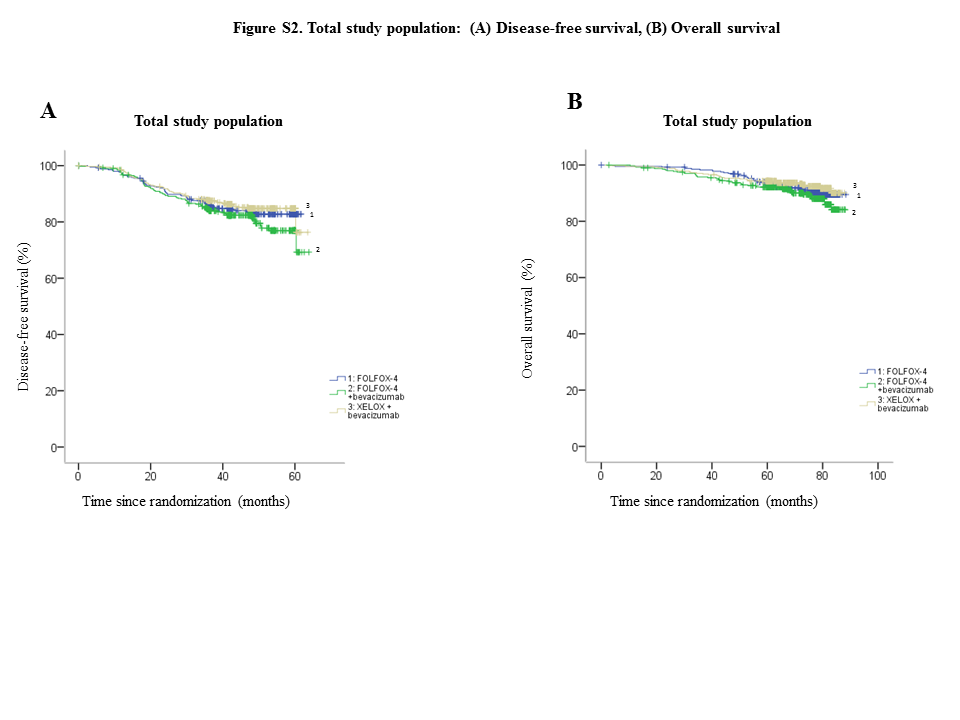

Supplement: Supplementary file 2 — Figure S2 [file 41416_2018_83_MOESM2_ESM.tif]
