# Supplementary material for: Predictive potential of tumour-stroma ratio on benefit from adjuvant bevacizumab in high-risk stage II and stage III colon cancer
Source: Br J Cancer. 2018 May 14;119(2):164–9. doi: 10.1038/s41416-018-0083-0 (PMC6048031; doi:10.1038/s41416-018-0083-0)
Supplement: Supplementary file 5 — Appendix 1 [file 41416_2018_83_MOESM5_ESM.docx]

**Appendix 1**

**Detailed description tumour stroma (TSR) scoring procedure**The TSR is evaluated using conventional microscopy on 5µm thick H&E stained formalin-fixed paraffin-embedded tissue slides from the primary tumour. The intra-tumoural stroma formation is assessed at the most invasive part of the tumour, since this area is most influential for tumour progression. This was determined in a study in which multiple H&E slides from different tumour areas were available for evaluation. Throughout the tumour there is heterogeneity in the stroma percentage, with the highest stroma percentages in areas with the deepest tumour invasion in the bowel wall (higher T-stage). [18] For retrospective studies, the tissue slide containing the most invasive part of the tumour is used for TSR evaluation because this generally corresponds best to the tissue slide used for routine pathological examination of the T-status as indicated in the pathology report. If not available, all available tumour slides are collected and analysed.

Using a 2.5x or 5x objective the area with the highest amount of stroma is selected. Subsequently, using a 10x objective, only those image fields are scored where neoplastic cells are present at all borders. Scoring percentages are given per 10-fold increments (10%, 20% et.) per image field. Identifying a single image-field with high stroma content is decisive for a final stroma classification. A statistically determined cut-off value of 50% has a maximum discriminative power and distinguishes between stroma-high (>50%) and stroma-low (≤50%) patients. [18]
